# The Effects of Radiation and Dose-Fractionation on Cancer and Non-Tumor Disease Development 

**DOI:** 10.3390/ijerph9124688

**Published:** 2012-12-18

**Authors:** William Liu, Benjamin M. Haley, Mary J. Kwasny, Jian-Jian Li, David J. Grdina, Tatjana Paunesku, Gayle E. Woloschak

**Affiliations:** 1 Department of Radiation Oncology, Northwestern University Feinberg School of Medicine, Chicago, IL 60611, USA; E-Mails: wliu@u.northwestern.edu (W.L.); benjamin.haley@gmail.com (B.M.H.); tpaunesku@northwestern.edu (T.P.); 2 Department of Preventative Medicine, Northwestern University Feinberg School of Medicine, Chicago, IL 60611, USA; E-Mail: m-kwasny@northwestern.edu; 3 Department of Radiation Oncology, The University of California Davis, Sacramento, CA 95817, USA; E-Mail: jian-jian.li@ucdmc.ucdavis.edu; 4 Department of Radiation and Cellular Oncology, The University of Chicago, Chicago, IL 60637, USA; E-Mail: dgrdina@uchicago.edu

**Keywords:** radiation induced toxicities, ionizing radiation, radiation-induced tumors

## Abstract

The Janus series of radiation experiments, conducted from 1970 to 1992, explored the effects of gamma and neutron radiation on animal lifespan and disease development. Data from these experiments presents an opportunity to conduct a large scale analysis of both tumor and non-tumor disease development. This work was focused on a subset of animals from the Janus series of experiments, comparing acute or fractionated exposures of gamma or neutron radiation on the hazards associated with the development of tumor and non-tumor diseases of the liver, lung, kidney or vascular system. This study also examines how the co-occurrence of non-tumor diseases may affect tumor-associated hazards. While exposure to radiation increases the hazard of dying with tumor and non-tumor diseases, dose fractionation modulates these hazards, which varies across different organ systems. Finally, the effect that concurrent non-cancer diseases have on the hazard of dying with a tumor also differs by organ system. These results highlight the complexity in the effects of radiation on the liver, lung, kidney and vascular system.

## 1. Introduction

Epidemiologic studies of radiation exposure on human populations have been limited to non-experimental ecological, cohort, and case-control studies. These studies have largely focused on radiation workers [1,2], individuals accidentally exposed to radiation through nuclear accidents (Chernobyl [3]), or atomic bomb survivors [4]. Because of the retrospective nature of many of these studies, dose estimates in human studies are often not exact. Naturally, due to ethical considerations, lifetime studies involving experimental, rigorously controlled conditions as well as acute and chronic radiation exposures, could only be done with animal models. Thus, the analysis of controlled animal data, when considered along-side non-experimental epidemiological studies in humans, may allow for a more accurate picture of the effects of radiation on disease. 

Patients exposed to radiation in the medical context are of particular concern, where directed radiation treatments of tumors could also result in off-target effects in neighboring tissues. For example, the use of radiotherapy in the treatment of breast cancer, typically in combination with the surgical excision of the tumor, is common. However, there have been studies suggesting that there exists a link between cardiovascular based mortality and morbidity after radiation exposure in patients with breast cancer [2]. This is due, presumably, to the proximity of the radiation treatment field to the heart and critical veins/arteries. The development of pulmonary diseases have also been associated with radiation therapy, particularly radiotherapies directed to the lungs [5]. Radiation pneumonitis is a disease which occurs in 5–15% of lung cancer patients who receive radiotherapy [6], and is commonly observed in both human and animal models [7]. Other non-tumor diseases, including liver disease [8] and renal disease [9] also could be affected by locally administered radiation (either to the liver or to the kidney). In addition to non-tumor diseases, secondary tumors can also develop as a consequence of radiation therapy [10,11]. 

The source of data for this work is the Janus series of radiation biology experiments, a large scale study consisting of over 49,000 mice, conducted continuously between 1970 through 1992 [12]. This dataset, which is currently hosted at Northwestern University (http://janus.northwestern.edu), is publically available, and presents a unique opportunity to explore the effects of radiation on animals (at a scale no longer considered feasible by today’s standards) in a controlled environment and under experimental conditions with a radiation source that is no longer available. Although some recent studies examining the Janus database [13] have aimed to re-discover and preserve this and other large scale radiation experiments [14,15], this manuscript is a result of a new effort to re-visit and analyze the data on mice exposed to total doses in the range of clinical exposures either acutely or in 60 fractions. Previous studies using this database either focused on the tumorigenic effects of radiation [16,17], or examined pathologies at lower total doses of radiation, comparing frequencies of disease between groups of control and irradiated animals [18]. 

This analysis was designed to investigate the effect of acute or fractionated exposure of gamma or neutron radiation on the hazards associated with the development of non-cancer diseases or cancer in four selected organ systems. In total, 2,785 gamma radiation exposed mice, 1,897 neutron irradiated animals and 1,740 controls were selected for this work; of these, 4,797 animals in total developed at least one of the diseases of interest. All diseases under investigation were limited to only four organ systems: renal, pulmonary, vascular, and liver; the development of tumors as well as the incidence of non-tumor diseases was recorded in both genders. These four organ systems have varying levels of radiation sensitivity, yet their dysfunction has significant systemic consequences. Additionally, this work examines the effect of non-tumor diseases on cancer related life-shortening. 

Ultimately, it is hoped that by examining these issues of cancer and non-tumor related hazards using experimental animal data, associations can be found in other data examining risks in humans, and thus act as a validation tool for future studies. 

## 2. Materials and Methods

### 2.1. Study Population

This analysis utilized historical data from the previously described Janus database [14]. Each observation in the database was associated with a radiation dose, fractionation, and quality, expressed as irradiation codes (specific dose values are also available), lifespan, date of first dose, proctor (pathologist) who performed the necropsy, as well as observed pathologies (the probable cause of death and accompanying diseases). 

During the course of the Janus experiments, observed gross pathologies were recorded and designated as “lethal” or “non-lethal”. Each animal was assigned a single (lethal) probable cause of death, but could have multiple additional “non-lethal” pathologies. In this analysis, both “lethal” and “non-lethal” variants of a particular pathology were combined, to form an overall pathology outcome for each animal. We favored this approach primarily because of the inherent subjectivity in assigning a probable cause of death, and that the proctors were under the requirement to assign a single most probable cause of death to each animal, thus precluding the possibility of assigning multiple organ dysfunction (MODS) as a cause of mortality [19]. Occasionally, a designation of “cause of death unknown” was assigned to cases where a probable cause of death could not be ascertained. The latter animals were excluded from this analysis because of the possibility that some of the pathologies were not recorded in such cases. 

This analysis has used a subset of the entirety of the Janus database, in order to use comparable radiation doses across different fractionation patterns. Mice from experiments JM-2 (Janus-2), JM-3, and JM-13 were used. Experiments JM-2 and 3 administered single doses of varying levels of gamma (90–600 cGy) or neutron radiation (13.5–40 cGy), while in experiment JM-13, mice were irradiated in sixty fractions at a closely matched total dose of gamma or neutron radiation. Overall, this analysis uses data collected on a total of 6,422 mice; 2,785 mice exposed to gamma radiation, 1,897 exposed to neutron radiation, and 1,740 controls sham exposed once or sixty times. All radiation was delivered as whole body exposures. 

This analysis used both male and female lab mice (*M. musculus*), since the outcomes of interest involved organ systems that are not sex specific. These were F1 animals from a cross between C57BL/6 females and BALB/c males, from parent stocks maintained throughout the twenty years of data accumulation. Control and experimental animals were all kept in a common environment in accordance with contemporary animal care protocols over the course of the study. 

### 2.2. Data Collection

All mice in this archive were irradiated for the first time at an average age of 112 days (SD: 4.35 days) using the Janus reactor, a 200 kW uranium fueled reactor. The first experiments were conducted in 1972 (JM-2) with continuous experimentation and data collection occurring over a decade, with the final animal autopsied in 1984 (JM-13). For mice receiving a fractionated exposure, each fractionated dose was given once weekly over sixty weeks; thus, the age difference between animals at the conclusion of irradiation amounted to a whole year. 

After irradiation, mice were checked for death twice daily, seven days a week, 365 days per year. Necropsies were performed by proctors, both on mice which were found dead and moribund animals which were then euthanized. The name of the proctor, as well as accompanying information (*i.e.*, date of death, type of radiation used, *etc.*) were recorded, along with pathology observations. All mice were tracked until death (natural death or moribund), with the age at death recorded. 

Since the original Janus experiments grouped recorded gross pathologies by disease types, the same designations were used here as well. The pathologies cholecystitis, cirrhosis, and hepatitis were grouped under liver diseases. Emphysema, lobar pneumonia, pneumonitis, and pneumonia were classified as pulmonary diseases. Myocardium pericardium, and thrombus were classified as cardiovascular diseases. Chronic renal disease, hydronephrosis, and polycystic kidney were classified as renal diseases. While lung and vascular tumors were each assigned their own group, renal system tumors consisted of urinary bladder, kidney, and miscellaneous urogenital tumors (excluding tumors of the gonads and reproductive organs), while liver tumors consisted of both liver and gallbladder tumors. These eight overall pathology groupings were used as event indicators at the time of death in this analysis. Outcomes were binary—either an animal had an observed pathology at death or it did not. Mice with non-tumor diseases other than the diseases mentioned above were also included under an “other non-tumor disease” outcome, while mice with tumors other than the aforementioned tumors were included under the “other tumor” outcome. 

All tissue samples from each mouse were preserved through paraffin embedding—these tissues are still available and hosted by Northwestern University, as the Janus Tissue Archive [20]. After pathological evaluation, these records were then entered into a database, first hosted on an IBM mainframe, then in an Oracle database, and now as a mySQL database hosted at Northwestern University (janus.northwestern.edu). 

Over the years, the pathology data in this dataset have been verified via independent audit, with over 90% of pathological observations made during the course of each study confirmed through a re-evaluation of the preserved tissues [12]. 

### 2.3. Statistical Analysis

Statistical analyses were performed using R 2.9.2 (www.r-project.org), with a significance level of 0.05 used throughout the analysis. 

The following exposures and covariates were examined in this analysis: lifespan (continuous), sex (dichotomous), type of radiation dose (categorical: control, gamma, or neutron), total radiation dose (continuous), and fractionation (dichotomous: single fraction *vs.* sixty fractions). Since neutron radiation is a densely ionizing radiation, and thus of different quality than gamma radiation at an equal dose (Gy), it was determined that neutron and gamma irradiated animals would be stratified into separate models. 

The total dose of radiation was characterized as a continuous variable, with discrete values of radiation exposure ranging from 90–600 cGy for gamma radiation, and 13.5–40 cGy for neutron radiation. In order to better model radiation using both linear and quadratic terms [21], doses were centered around the mean, by subtracting the mean gamma or neutron radiation dose from each observation. 

Separate Kaplan-Meier survival curves were generated for animals given either an acute or fractionated dose of gamma or neutron radiation. Animals were divided into separate dose groups, with animals given the highest (569–600 cGy for gamma, 40 cGy for neutron) or lowest (90–143 cGy for gamma, 20–21.5 cGy for neutron) doses analyzed together, while fractionated and acute doses within these ranges were matched as closely as possible. Animals given 143 cGy or 90 cGy non-fractionated gamma radiation were analyzed with animals given 100 cGy of fractionated gamma radiation to form the “lowest” gamma radiation dose group. Animals given 20 cGy of non-fractionated neutron radiation were analyzed with animals given 21 cGy of fractionated neutron radiation to form the “lowest” neutron radiation dose group. Animals given 569 cGy of non-fractionated gamma radiation were analyzed with animals given 600 cGy of fractionated gamma radiation to form the “highest” gamma radiation dose group. Finally, animals given 40 cGy of non-fractionated neutron radiation were analyzed with animals given 40 cGy of fractionated neutron radiation to form the “highest” neutron radiation dose group (to ensure the validity of grouping animals with similar total doses within a fractionation pattern, groupings were determined by log-rank tests of survival—doses of the same fractionation pattern were grouped only if there were no significant differences in survival). These KM curves used survival times for animals which died with the specific disease of interest. Animals were censored if an animal died with a disease other than the disease being studied. These curves show the difference in survival associated with having a particular disease, comparing animals given a fractionated radiation dose to a non-fractionated dose at a closely matched total dose.

Additionally, Cox proportional hazards regressions were run examining the effect of radiation on the hazard of dying with renal, liver, cardiovascular, or pulmonary disease (non-tumor diseases) or a renal system, liver, vascular, or lung tumor (cancer-diseases). These models were stratified by dose fractionation pattern—separate models were generated using animals given either an acute or fractionated radiation dose. The radiation dose was initially assumed to have a linear-quadratic relationship with hazard, but in cases where the quadratic term was found to be non-significant, only the linear term was included. The use of a simple linear radiation dose term to describe life-shortening was found to be sufficient in previous studies which compared multiple models [16,22]. Due to differences in the range of doses between gamma and neutron radiation models, gamma radiation doses which were originally in cGy were expressed in Gy, while in neutron radiation models, cGy was converted to 10 cGy “units” (*i.e.*, a one unit increase in neutron radiation dose can be interpreted as a 10 cGy increase). This was done in order to generate more easily interpretable regression coefficients. Dichotomous variables for both cancer and non-tumor disease were used as the event indicators. Sex was used as a (dichotomous) covariate in the adjusted regressions. 

Lastly, to examine the association between non-tumor and tumor disease at the time of death, hazards were estimated using sex adjusted, fractionation-stratified Cox regressions (using each cancer endpoint as an event indicator and the presence of each non-tumor disease as a dichotomous variable). Evaluation of the violation of the proportional hazards assumption for each Cox model was performed in R via a test for a non-zero slope in the regression of time on scaled Schoenfeld residuals [23]. 

## 3. Results

Table 1 presents an excerpt of the Janus dataset which was used in this study, with overall and stratified counts/percentages. The data used in this study were: (1) animals acutely exposed to total dose exposures close to the doses used in a clinical setting, (2) the animals that received a closely matched total dose in 60 weekly fractions and (3) the sham irradiated controls. 

**Table 1:** Data summary.

	Total	Gamma	Neutron	Control
Total #	6,422	2,785	1,897	1,740
Sex % (#)				
Male	55.33 (3,553)	57.70 (1,607)	55.35 (1,050)	51.49 (896)
Female	44.67 (2,869)	42.30 (1,178)	44.65 (847)	48.51 (844)
Fractions % (#)				
1	35.57 (2,284)	34.94 (973)	32.47 (616)	39.94 (695)
60	64.43 (4,138)	65.06 (1,812)	67.52 (1,281)	59.77 (1,045)

Lifespan days (mean, sd)	(934.69, 194.26)	(923.99, 198.52)	(909.49, 187.83)	(979.28, 186.83)
Non-tumor Outcomes % (#)				
Renal	8.08 (519)	6.64 (185)	6.75 (128)	11.84 (206)
Liver	1.12 (77)	1.18 (33)	1.32 (25)	1.09 (19)
(Cardio)vascular	0.965 (62)	0.90 (25)	0.63 (12)	1.44 (25)
Pulmonary	40.52 (2,602)	40.40 (1,125)	39.69 (753)	41.61 (724)
Other	60 (3,851)	59.93 (1,669)	58.46 (1,109)	61.67 (1,073)
Cancer (tumor) Outcomes % (#)				
Renal	1.46 (94)	0.90 (25)	3.22 (61)	0.46 (8)
Liver	5.34 (343)	4.78 (133)	6.59 (125)	4.89 (85)
Vascular	8.44 (543)	9.05 (252)	9.07 (172)	6.78 (118)
Pulmonary	42.34 (2,719)	41.62 (1,159)	42.49 (806)	43.33 (754)
Other	68.84 (4,421)	70.34 (1,959)	67.84 (1,287)	67.53 (1,175)

Baseline characteristics and outcomes of Janus experiments 2, 3, and 13—stratified by radiation quality. Values within parentheses for lifespan measures indicate the mean and standard deviation, while the remaining values are percentages indicating the proportion of cases in that particular treatment group. Values in parentheses are counts of cases.

The total doses under consideration were from 0.9 to 6 Gy for gamma rays or 0.135 to 0.4 Gy for neutron doses. These ranges were selected due to the RBE for fission spectrum neutrons used in the Janus experiments, which was believed to be at least equal to 10 [12]. 

The distribution of subjects across treatment groups varied when compared using sex and fractionation pattern, with males (N = 3,553) outnumbering females (N = 2,869). With respect to fractionation pattern, 2,284 animals were given a single treatment compared to 4,138 animals given a fractionated irradiation or sham-irradiation. 

**Figure 1:**
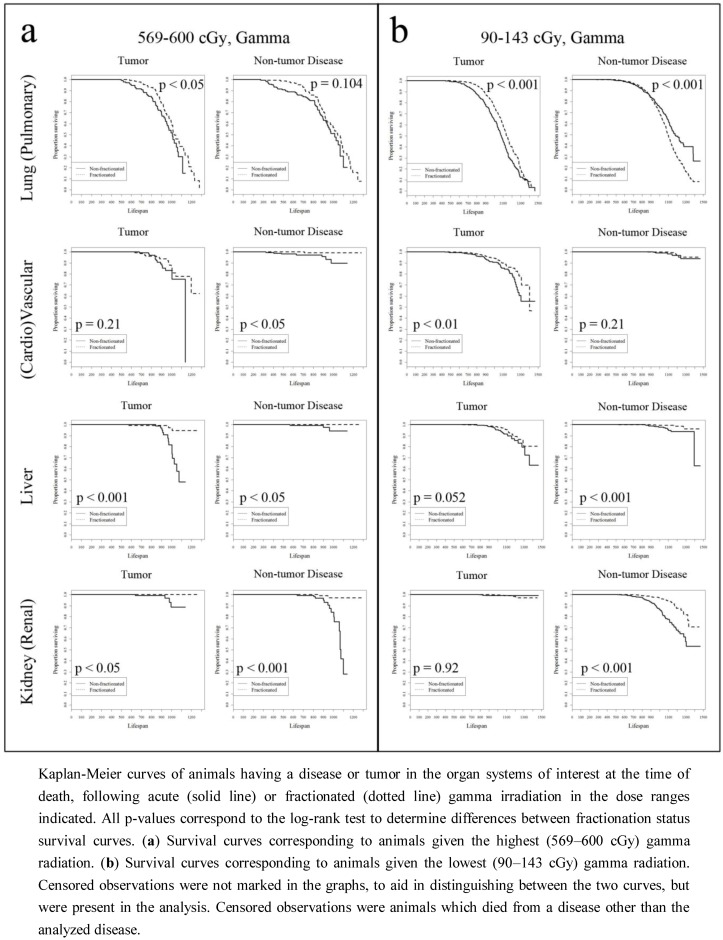
Gamma radiation survival curves.

**Figure 2:**
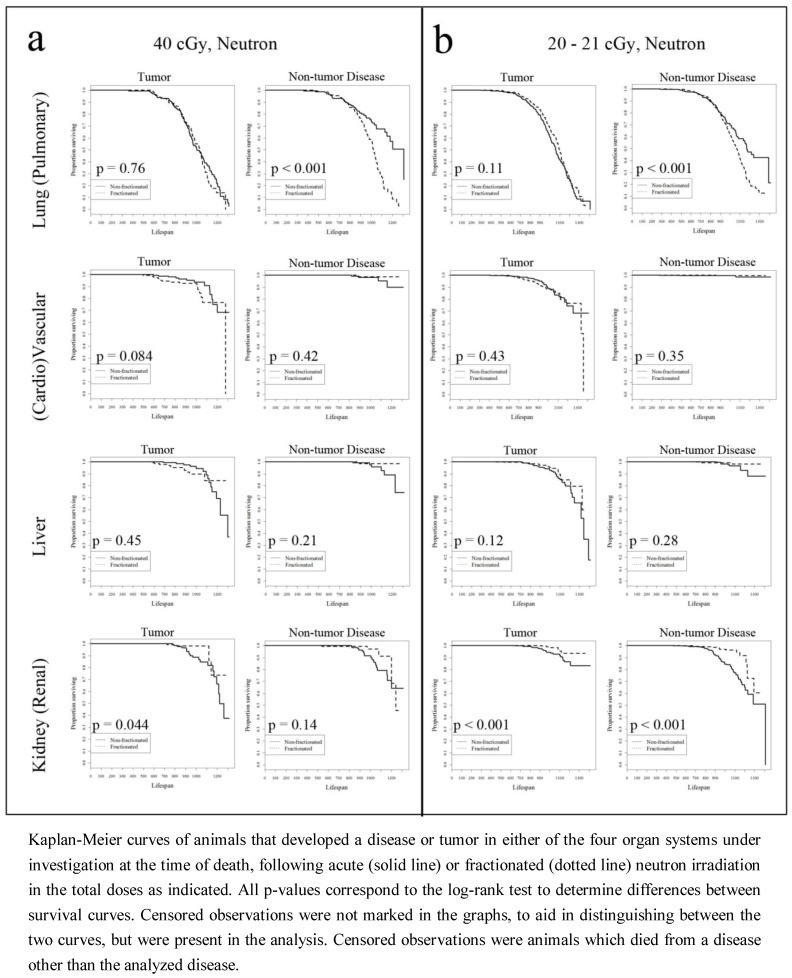
Neutron radiation survival curves.

Using the animals selected in Table 1, Kaplan-Meier (KM) survival curves focusing on the cancer and non-cancer diseases of the four organ systems also presented in Table 1 were constructed. These KM survival curves compared mice which received an acute dose of radiation, compared to mice which received a closely matched fractionated dose. These results are presented in Figure 1 and Figure 2. Figure 1(a) presents animals given 569–600 cGy of gamma radiation, using the presence of each disease or tumor at death as an event indicator. While it was difficult to interpret some of the results with low event counts, e.g., incidences of kidney, liver, or cardiovascular disease for animals given a fractionated dose, the general trend is that animals given a fractionated dose had a significantly lower hazard of having lung tumors, vascular tumors, and liver tumors at the time of death, compared to animals given a single radiation dose. This trend was also observed in the non-tumor diseases, with the exception of pulmonary disease. While these trends were again observed at the lowest range of gamma radiation doses (90–143 cGy gamma rays), the difference in life shortening related to pulmonary disease between fractionated and single exposures becomes significant, and interestingly, reversed—at 90–143 cGy gamma radiation, dose fractionation is associated with an increased hazard of dying with pulmonary disease. 

Figure 2(a,b) present Kaplan-Meier survival curves in animals given neutron radiation. As was observed in the animals exposed to gamma radiation, low counts of events in animals for a number of disease outcomes affected the analysis. However, mice exposed to fractionated doses had a higher hazard associated with pulmonary disease development compared to their acute exposure counterparts for both the highest and lowest neutron radiation treated animals, while the opposite was observed in the survival curves examining kidney related tumor and disease, particularly at the lowest neutron radiation dose. 

To test how gamma radiation and fractionation affect the hazard of dying with either a tumor or non-tumor disease, Cox proportional hazards regressions were generated. Table 2 presents adjusted (by sex) and unadjusted estimated hazard ratios examining gamma radiation, stratified by fractionation, and separated by organ system and further by tumor/non-tumor disease. The presented values are the estimated hazard ratios for radiation dose. Significant hazard ratios for the linear form of these regressions were found for many adjusted and unadjusted tumor models. It is important to note that a direct comparison in hazard ratios across fractionation patterns should not be made, since both fractionation patterns have varying underlying hazard functions. One can only examine increases in hazard in response to dose, relative to each fractionation pattern. For example, in the adjusted models, for each 1 Gy increase in gamma radiation total dose, the relative increase in the hazard of dying with a vascular tumor was 19% for fractionated exposures, while the relative increase in hazard was 22% for acute exposures. Thus, the relative effect of increasing gamma radiation dose on the hazard of dying with a vascular tumor varied between mice given fractionated or acute irradiation.

In the adjusted models for renal system tumor related hazard, only the animals irradiated with a single dose had a significant hazard ratio, with a 1 Gy increase in gamma radiation dose corresponding to a 70% increase in renal system tumor related hazard. Interestingly, the relative increase in the hazard of dying with a lung tumor for a 1 Gy increase in gamma radiation was at 15% in animals exposed to 60 weekly fractions, while this increase was at 11% in animals given a single radiation dose. No significant quadratic relationships were found between tumor related hazard and dose. There were fewer significant hazards of non-tumor diseases, and therefore fewer differences across fractionation groups. For example, the liver disease related hazard was significantly increased (by 34% in the adjusted model) in animals given a single dose, while a 13% increase in lung disease related hazard was observed in animals given fractionated irradiation. Interestingly, a significant quadratic relationship was observed between kidney disease related hazard and dose, but only in the un-adjusted model. 

**Table 2:** Risk of organ system pathologies, gamma radiation.

Organ	Model	Fractions	Tumor		Non-tumor Disease	
			Linear	Quadratic	Linear	Quadratic
			HR (95% Conf. Int)	HR (95% Conf. Int)	HR (95% Conf. Int)	HR (95% Conf. Int)
Kidney (renal)	Adj.	60	1.11 (0.798, 1.56)	-	1.00 (0.874, 1.15)	-
		1	**1.71 (1.36, 2.17)**	-	0.931 (0.797, 1.09) *	1.07 (1.01, 1.13) *
	Unadj.	60	1.11 (0.80, 1.56)	-	0.987 (0.859, 1.13)	-
		1	1.73 (1.38, 2.18) *	-	**0.722 (0.626, 0.834)**	**1.15 (1.09, 1.22)**
						
Liver	Adj.	60	1.03 (0.899, 1.19)	-	0.846 (0.552, 1.30)	-
		1	**1.40 (1.27, 1.55)**	-	**1.34 (1.10, 1.62)**	-
	Unadj.	60	1.04 (0.90, 1.20)	-	0.846 (0.551, 1.30)	-
		1	**1.39 (1.26, 1.55)**	-	1.37 (1.13, 1.65)	-
						
Vascular (cardio)	Adj.	60	**1.19 (1.11, 1.28)**	-	0.941 (0.675, 1.31) *	-
		1	**1.22 (1.12, 1.35)**	-	0.674 (0.397, 1.14)	**1.3 (1.09, 1.56)**
	Unadj.	60	**1.19 (1.10, 1.29)**	-	0.95 (0.68, 1.32)	-
		1	**1.23 (1.12, 1.35)**	-	0.697 (0.413, 1.17)	**1.29 (1.08, 1.54)**
						
Lung (pulmn.)	Adj.	60	**1.15 (1.12, 1.20)**	-	**1.13 (1.09, 1.17)**	-
		1	**1.11 (1.07, 1.17)**	-	1.04 (0.944, 1.14)	**1.05 (1.02, 1.09)**
	Unadj.	60	**1.16 (1.12, 1.21)**	-	**1.12 (1.09, 1.16)**	-
		1	**1.13 (1.09, 1.19)**	-	1.17 (1.11, 1.23)	**1.06 (1.02, 1.09)**

Table of Cox regression hazard ratios (corresponding to the gamma radiation dose term) and accompanying 95% confidence intervals in gamma radiation models. Values in bold indicate significant hazard ratios at the 5% level. Models with non-significant quadratic terms were fit using only the linear term, and those estimates are presented here. Quadratic HR estimates are presented only for models in which there was a significant quadratic term. * indicates that the particular model did not satisfy the proportional hazards assumption.

We next tested how neutron radiation and fractionation affected the hazard of dying with a tumor/non-tumor disease. Table 3 presents sex adjusted and unadjusted estimated hazard ratios examining neutron radiation, stratified by fractionation, and separated by organ system and then by tumor/disease. Significant hazard ratios for the linear components of these regressions were found for nearly all adjusted and unadjusted tumor models. For example, in the adjusted models, for a 0.01 Gy increase in neutron radiation dose, the relative increase in the hazard of dying with a renal system tumor was 96% in animals given sixty fractions, while the relative increase in hazard was 375% in animals given a single dose. A number of non-tumor diseases were also found to be significantly associated with the linear component of total radiation dose. For example, with a 0.01 Gy increase in neutron radiation dose, there was a 24% increase in the hazard of dying with a renal disease (in the fractionated irradiation, adjusted model), a 43% increase in liver disease associated hazard (in the acute irradiation, adjusted model), and a 19% increase in pulmonary disease associated hazard (in the fractionated irradiation, adjusted model). 

**Table 3:** Risk of organ system pathologies, neutron radiation.

Organ	Model	Fractions	Tumor		Non-tumor Disease	
			Linear	Quadratic	Linear	Quadratic
			HR (95% Conf. Int)	HR (95% Conf. Int)	HR (95% Conf. Int)	HR (95% Conf. Int)
Kidney (renal)	Adj.	60	**1.96 (1.45, 2.66)**	-	**1.24 (1.05, 1.45)**	-
		1	**3.75 (2.19, 6.42)**	**0.782 (0.634, 0.965)**	1.02 (0.896, 1.15)	**1.11 (1.00, 1.23)**
	Unadj.	60	**1.98 (1.47, 2.69)**	-	**1.18 (1.01, 1.39)**	-
		1	**3.67 (2.15, 6.27)**	**0.769 (0.626, 0.946)**	**0.892 (0.800, 0.993)**	-
						
Liver	Adj.	60	**1.40 (1.22, 1.60)**	-	1.10 (0.767, 1.58)	-
		1	**1.24 (1.07, 1.44)**	-	**1.43 (1.07, 1.91)**	-
	Unadj.	60	**1.41 (1.23, 1.61)**	-	1.09 (0.762, 1.56)	-
		1	**1.24 (1.08, 1.43)**	-	**1.45 (1.10, 1.89)**	-
						
Vascular (cardio)	Adj.	60	**1.24 (1.12, 1.38)**	-	0.753 (0.469, 1.21)	-
		1	**1.30 (1.11, 1.52)**	-	1.05 (0.746, 1.49)	-
	Unadj.	60	**1.25 (1.13, 1.39)**	-	0.747 (0.466, 1.20)	-
		1	**1.21 (1.05, 1.39)**	-	1.09 (0.782, 1.54)	-
						
Lung (pulmn.)	Adj.	60	1.23 (1.17, 1.30) *	-	**1.19 (1.14, 1.25)**	-
		1	1.05 (0.993, 1.11)	-	0.992 (0.919, 1.07) *	-
	Unadj.	60	**1.25 (1.19, 1.32)**	-	**1.19 (1.13, 1.24)**	-
		1	**1.09 (1.03, 1.15)**	-	0.983 (0.913, 1.06) *	-

Table of Cox regression hazard ratios (corresponding to the neutron radiation dose term) and accompanying 95% confidence intervals in neutron radiation models. Bolded values indicate significant hazard ratios at the 5% level. Models with non-significant quadratic terms were fit using only the linear term, and those estimates are presented here. Quadratic HR estimates are presented only for models in which there was a significant quadratic term.* indicates that the particular model did not satisfy the proportional hazards assumption.

Next, we examined how the presence of a non-tumor disease affects the risk of dying with a tumor following acute or fractionated irradiation. Table 4 presents estimated hazard ratios of dying with both a tumor and a non-tumor disease, adjusted for total dose, sex, and stratified by fractionation. Both positive and negative associations were found. In the models where animals were given fractionated gamma radiation, the relative decrease in the hazard of dying with a lung tumor with concomitant cardiovascular disease was ~70% compared to animals without cardiovascular disease, while the relative decrease in hazard(with the same conditions) for acutely irradiated mice was ~55%. The hazard of dying with a vascular tumor and a renal disease decreased by ~64% in animals given a fractionated radiation dose, compared to animals without a renal disease, while a relative decrease of ~46% was found in animals given a single dose of radiation. Similarly, among animals given sixty fractions, there was a ~61% decrease in the hazard of dying with a vascular tumor and a pulmonary disease compared to mice without a pulmonary disease, while the relative decrease in hazard was ~41% in animals given a single fraction.

In animals given neutron radiation, a ~39% decrease in the hazard of dying with a lung tumor and a renal disease was observed in animals given a fractionated dose, compared to animals which died with a lung tumor without renal disease, while a relative decrease in hazard of 42% was found in animals given a single dose. Other disease models with a significant decrease in the hazard of co-occurrence after neutron exposure were the pairs (i) pulmonary disease and renal system tumor hazard, (ii) pulmonary disease and liver tumor hazard, (iii) renal disease and vascular tumor hazard, and (iv) pulmonary disease and vascular tumor hazard (Table 4). Unsurprisingly, in the models where animals were given either qualities of radiation, pulmonary disease was significantly associated with an increase in the hazard of dying with a lung tumor, across both fractionation patterns.

**Table 4:** The effect of disease on tumor risk.

Tumor	Disease	Gamma		Neutron	
		Fractionated	Non-Fractionated	Fractionated	Non-Fractionated
		HR (95% Conf. Int)	HR (95% Conf. Int)	HR (95% Conf. Int)	HR (95% Conf. Int)
Kidney (renal)	Renal	-	1.50 (0.473, 4.74)	0.539 (0.072, 4.04)	0.603 (0.235, 1.55)
	Liver	-	-	-	0.459 (0.063, 3.35)
	Cardio (vascular)	-	2.58 (0.332, 20.1)	-	1.31 (0.179, 9.62)
	Pulmonary	**0.184 (0.0415, 0.819)**	0.703 (0.230, 2.14)	**0.238 (0.080, 0.705)**	1.13 (0.572, 2.25)
					
Liver	Renal	0.370 (0.0904, 1.51)	0.682 (0.401, 1.16)	0.570 (0.231, 1.41)	0.610 (0.340, 1.09)
	Liver	1.82 (0.253, 13.09)	0.587 (0.144, 2.39)	0.782 (0.109, 5.63)	0.557 (0.138, 2.27)
	Cardio (vascular)	1.13 (0.276, 4.61)	0.968 (0.238, 3.93)	0.752 (0.104, 5.42)	1.54 (0.378, 6.25)
	Pulmonary	0.820 (0.552, 1.22)	0.910 (0.60, 1.38)	**0.566 (0.382, 0.839)**	1.29 (0.830, 2.02)
					
Vascular (cardio)	Renal	**0.358 (0.132, 0.967)**	**0.533 (0.312, 0.912)**	**0.164 (0.0405, 0.662)**	1.08 (0.670, 1.74)
	Liver	0.926 (0.130, 6.61)	0.458 (0.113, 1.86)	NA	0.650 (0.160, 2.65)
	Cardio (vascular)	1.05(0.336, 3.31)	0.390 (0.0541, 2.77)	0.971 (0.240, 3.93)	0.808 (0.112, 5.81)
	Pulmonary	**0.389 (0.288, 0.525)**	**0.586 (0.385, 0.892)**	**0.400 (0.289, 0.554)**	**0.415 (0.230, 0.746)**
					
Lung (pulmn.)	Renal	0.919 (0.674, 1.25)	**0.608 (0.492, 0.751)**	**0.612 (0.437, 0.857)**	**0.577 (0.460, 0.725)**
	Liver	0.584 (0.188, 1.82)	0.802 (0.527, 1.22)	0.576 (0.258, 1.29)	0.595 (0.349, 1.01)
	Cardio (vascular)	**0.301 (0.125, 0.726)**	**0.448 (0.213, 0.944)**	0.501 (0.224, 1.12)	0.523 (0.217, 1.26)
	Pulmonary	**1.15 (1.02, 1.30)**	1.55 (1.35, 1.79) *	**1.15 (1.00, 1.31)**	1.75 (1.50, 2.04) *

Table of Cox regression hazard ratios and accompanying 95% confidence intervals in gamma and neutron radiation models corresponding to the estimated hazard ratio of the disease of interest. Models used to obtain these estimated HR coefficients used a linear dose term only, due to the lack of a significant quadratic dose term in the previous cancer based models. Bolded values indicate significant hazard ratios at the 5% level. * indicates that the particular model did not satisfy the proportional hazards assumption.

## 4. Discussion

With such a large and comprehensively documented database of controlled radiobiology experiments, the Janus series of experiments presents a rare opportunity to evaluate the pathological effects of radiation on animals. This manuscript examined the effects of both gamma and neutron irradiation on a set of well characterized biological endpoints: lung, vascular, liver, and renal system cancers and non-tumor pathologies of the same four organ systems (pulmonary, cardiovascular, liver, and renal disease). We also sought to analyze the effects of dose fractionation on these endpoints, and the association between non-tumor diseases and tumor associated hazard. 

This work shows that fractionation plays a significant role in both tumor and non-tumor disease related hazard, in both gamma and neutron irradiated animals. Kaplan-Meier curves presented in Figure 1 and Figure 2 indicated that fractionation was significantly associated with a longer survival across multiple diseases and tumors. The only exception was pulmonary disease, found in mice dying at earlier ages following fractionated exposure to either gamma rays or neutron radiation. This was found in mice exposed to both the highest and the lowest doses of neutron radiation, and only for the lowest doses of gamma rays (while the difference between KM survival curves of animals dying with pulmonary disease at the highest gamma radiation dose was not significant). Further, this relationship was not seen in the survival curves of mice with lung tumors. In these mice, fractionated gamma radiation was associated with pulmonary cancer occurrence in longer living animals than in their acutely exposed counterparts. While it was expected that the hazard of dying with a lung tumor would be lower in animals given a fractionated radiation dose, it was unexpected that the same relationship was not observed in animals dying with a non-tumor pulmonary disease. Because a correlation between the development of lung tumors and pulmonary disease (particularly pneumonitis) has been observed in humans [24] this result is indicative of the complexities of tumor formation and disease progression in response to total radiation dose and dose fractionation. 

Secondly, increases in total gamma and neutron radiation dose were both significantly associated with a higher hazard of having a tumor at the time of death, while the hazard of dying with a non-tumor disease was found to be significantly associated with increasing radiation exposure for only a few non-tumor diseases. This confirms the linear-dose response of tumor expression at the time of death for both gamma and neutron radiation [16]. More interesting, however, was of the relative lack of association between increasing radiation dose and non-tumor disease related hazard. For example, while the hazard of dying with a pulmonary disease was significantly increased as radiation dose was escalated (across both gamma and neutron radiation groups), a similar increase was observed only in certain liver and kidney related hazard models. A likely explanation for this observation is that many of these animals may have died from a cancer based disease before they had a chance to develop a non-tumor disease. Indeed, previous studies examining the Janus database have observed that roughly 85% of animals died from a neoplastic disease [16,25]. The effect of radiation on the hazard of dying with a non-tumor disease may be obscured by the large number of animals that died earlier due to a tumor. 

Thirdly, dose fractionation (both gamma and neutron radiation) had different relative effects on hazard across the different pathologies, with some pathology models (e.g., vascular tumors) having an increase in relative hazard when moving from non-fractionated to fractionated radiation, as the total radiation dose was escalated. Meanwhile, other pathologies (e.g., lung tumors) had a decrease in relative hazard, when moving from non-fractionated to fractionated radiation, as the total dose increased. While the baseline hazards varied between fractionated and non-fractionated animals, these relative changes in hazard in response to increased radiation reflect the varied effect that fractionation might have on a particular organ system. For example, in animals given sixty weekly fractions of neutron radiation, the hazard of a 0.01 Gy increase resulted in a 96% increase in the hazard of dying with a renal system tumor, while in acutely exposed animals, the dose-hazard relationship was found to be linear-quadratic, with the increase in hazard (from the linear component of the regression) found to be by 375%. This trend was reversed for the hazard of dying with a lung tumor, in both gamma and neutron irradiated animals. This varied response to dose fractionation again reflects the significant differences in the survival of animals afflicted by tumors of different organ systems. The lungs are particularly sensitive to dose fractionation, with radiation considered to primarily affect pulmonary endothelial cells [5]. These differences in the response to dose fractionation may also be indicative of the comparative radiosensitivity and rate of cellular turnover of the lung compared to the kidney or liver. 

Fourth, the simultaneous occurrence of non-tumor diseases with a tumor was significantly associated with changes in tumor related hazard, while again, these relative changes in hazard varied between models where animals were exposed to fractionated and non-fractionated irradiation. In Table 4, the presence of a pulmonary disease is significantly associated with an increased hazard of dying with a lung tumor, compared to animals without a pulmonary disease. However, the tumor related hazards of all other organ systems—kidney, liver, and vascular, decreased when in the presence of a variety of non-tumor diseases. This suggests that pathologies of the lung organ system (tumor and non-tumor disease) are inter-dependent on each other, an interdependence that is not observed in other organ systems. Further, relative decreases in hazard also varied across fractionation groups, indicating that fractionation also plays an important role in modulating the association between a particular disease and a particular tumor. 

This study has a number of caveats. First, since this analysis used animal data, the extrapolation of predictions to human subjects may be difficult. While previous studies have shown the usefulness of this Janus dataset in making cross-species predictions [25,26] more work must be done in determining which diseases may be comparable between species, with future studies focusing on Janus experimental data in larger mammals (dogs [26,27]), data which is also now publically available. Second, since this analysis included data from three experiments, it can be expected that the expansion of this analysis through the addition of other JM experiments could allow for more robust regression models, although the experiments (and doses) considered here were carefully selected to ensure close matches across fractionation patterns. 

Ultimately, while this investigation confirmed that increased radiation indeed is associated with an increased hazard of dying with cancer, it has also shown that radiation plays an important role in the hazards associated with dying with a non-tumor disease. Further, dose fractionation strongly modulates the effect of radiation on these hazards. Finally, the co-occurrence of a non-tumor disease can also affect the hazards associated with dying with a tumor, but varies across organ systems. The ability to examine these radiobiology questions in animals, using the data generated through a series of carefully controlled experiments, is a rare opportunity, and it is hoped that this work will further promote the use and analysis of the Janus database as a unique and valuable radiobiology resource. 
